# Association Between Neuronal Pentraxin 2, ADHD Symptoms, and Executive Functioning in Adults with ADHD: A case-control study

**DOI:** 10.1192/j.eurpsy.2025.732

**Published:** 2025-08-26

**Authors:** S. Kaya, A. Kandeğer, R. Kocabaş, A. E. Yorulmaz, B. Sağlıyan, Ö. Bayırlı

**Affiliations:** 1 Psychiatry; 2 Biochemistry, Selçuk University, Konya, Türkiye

## Abstract

**Introduction:**

Neuronal pentraxins (NPTXs), particularly NPTX2, play a key role in glutamate modulation and the stabilization of AMPA receptors, influencing synaptic plasticity. Studies have shown a positive correlation between NPTX2 levels and neurocognitive function in neurodegenerative diseases, and it has been proposed as a potential biomarker for synaptic degeneration (San José *et al.* J Neural Transm 2022; 129 207-230). However, the role of NPTX2 in the etiology of ADHD has not been explored in human samples.

**Objectives:**

This study aims to investigate the relationship between NPTX2 levels and ADHD symptoms in adults with ADHD, while also exploring the potential impact of NPTX2 on executive functions, which are frequently impaired in this population.

**Methods:**

Adults with ADHD who were medication-free and had no comorbid psychiatric diagnoses, along with a similar control group with no psychiatric diagnoses, were included in the study. All participants were diagnostically assessed using the Structured Clinical Interview for DSM-5 Disorders-Clinician Version. Participants completed measurements related to both ADHD and comorbid conditions. In addition, participants underwent a battery of neuropsychological tests, including the Stroop Test, Cancellation Test, Serial Digit Learning Test, Wisconsin Card Sorting Test, and Judgment of Line Orientation Test. The serum samples obtained after centrifugation were stored at -80°C until the time of analysis, at which point NPTX2 levels were measured using the ELISA method. Informed consent was obtained from all participants, who voluntarily agreed to participate. The study was approved by the Local Ethics Committee of Selçuk University under decision number 2023/495.

**Results:**

The study included 79 adults with ADHD and 70 healthy controls. Among the participants, 57.7% (n=86) were female, with a mean age of 23.50 ± 4.37 years. Both groups were comparable in terms of age, gender, total years of education, and body mass index. Individuals with ADHD showed higher levels of ADHD- and comorbidity-related symptoms, as well as poorer executive function profiles, compared to healthy controls. NPTX2 levels were significantly elevated in the ADHD group. Significant positive correlations between NPTX2 levels and clinical and neurocognitive data were observed in the ADHD group, but not in the control group. Finally, linear regression analyses conducted separately for each group revealed significant F values, showing that in adults with ADHD, NPTX2 levels were significantly associated with ADHD symptoms independent of age, gender, years of education, and anxiety/depression scores, whereas this relationship was not observed in healthy controls (Image 1).

**Image 1:**

**Figure:**
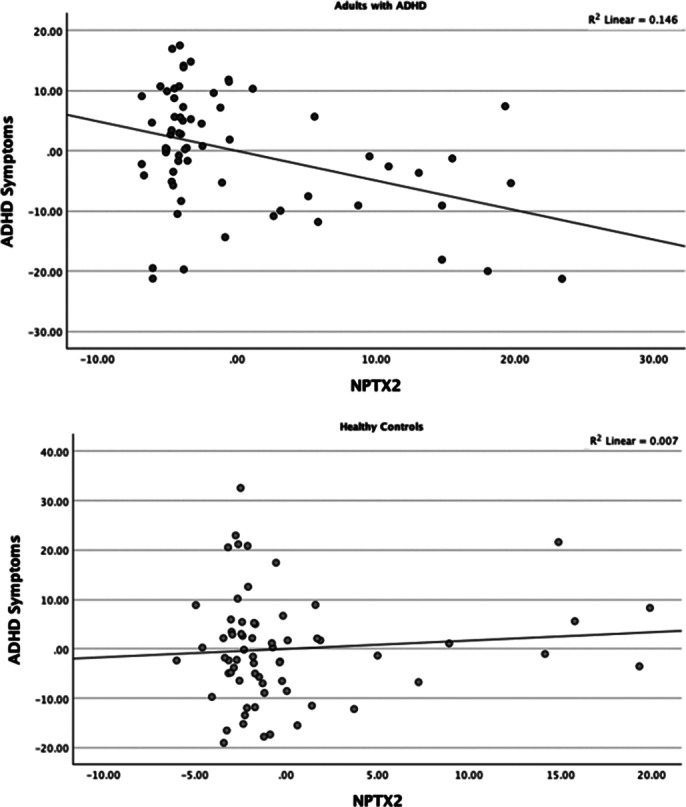

**Conclusions:**

These results highlight the need for further research into the role of NPTX2 and other neuronal pentraxins in ADHD and suggest that NPTX2 may serve as a biological marker for this disorder.

**Disclosure of Interest:**

None Declared

